# SLEEP OBSTRUCTIVE APNEA SYNDROME CONSEQUENCES

**DOI:** 10.1016/S1808-8694(15)30070-7

**Published:** 2015-10-19

Many paleontologists believe that between 200 and 300 thousand years ago, human ancestors started to suffer their most important anatomical change: the transformation of the upper airways. Such change included facial shortening, foramen magnus anteriorization, angle reduction between the horizontal and vertical portions of the upper airway, and especially larynx descending. This new configuration, with a longer and narrower supralaryngeal airway, facilitated the development of speech and language, being paramount for the success of a human creativity boom that occurred 40 thousand years ago and turned us into the dominating species in the planet.

Nonetheless, history tells us that nature always charges a price for evolution. This new airway anatomy gave more respiratory relevance to our pharynx, turned into a vulnerable frameless segment and thus, prone to collapse during inspiration, especially during sleep. This led to the first roots of the sleep obstructive apnea syndrome (SOAS), which happens only to humans among mammals, except for the hunting British Bulldogs (brachycephalic animals).

Medical attention towards SOAS diagnosis and its consequences has been growing. Polysomnography alterations matching those seen in SOAS are present in 1 to 2% of children and, among adults, in 24% of men and 9% of women. In the age range between 50 and 60 years, such alterations happen in 31% of men and 16% of women.

The consequences of SOAS among children are well known by otolaryngologists. Those snoring children with apnea, night time hypoxemia and sleep fragmentation may present with low school performance, attention deficit and hyperactivity. Moreover, these children have low height-weight gain and suffer from changes in their facial skeleton development. Less remembered are the cardiovascular alterations, such as pulmonary hypertension and, in more severe cases, cor pulmonale. Differently from adults, the association between systemic arterial hypertension and SOAS in children still is controversial.

Now, in adults with SOAS, the respiratory and neurological alterations that occur during sleep may bring about consequences that science has proven to be increasingly more numerous. These include memory loss, impairment in acquiring new information; attention reduction, with a greater risk of being involved in accidents; greater irritability and emotional lability. SOAS may also cause inflammatory, endothelial, atherosclerotic, autonomic and metabolic alterations, thus increasing the person’s risk of developing systemic arterial hypertension, cardiac arrhythmias, coronary arteries’ disease and congestive heart failure. It is still unclear if there is any association between SOAS and stroke.

Moreover, oxidative stress resulting from hypoxemia/reoxygenation events has been associated with an increase in peripheral insulin action resistance, and also increase in erythrocyte settling rate (ESR), C-Reactive Protein, Interleukin 6 and tumor necrosis factor alpha (inflammatory markers). The reduction in deep sleep (delta sleep) is associated with a reduction in growth hormone secretion rate. About 25% of male patients with SOAS have libido reduction or sexual impotence.

The effective treatment of SOAS may revert or damp some of these consequences while others are irreversible, emphasizing the need for the early treatment of this syndrome.

SOAS investigation is part of the basic admission questionnaire answered by patients in USA hospitals. A confirmed diagnosis or only a SOAS suspicion may cause a patient to be transferred from a surgical center to a better equipped facility.

SOAS also brings consequences to anesthesia in surgical cases. By principle, pre-anesthetic sedation must not be done, and it is recommended to avoid benzodiazepine agents, reduce the use of opioid and intensively monitor these cases for at least two hours after interrupting the administration of anesthetics, securing the patient’s alert status and respiratory comfort.

With life span increasing in our population, it is likely that the cumulative morbid impact SOAS may have be increasingly more apparent. All health care professionals will have to participate in the prevention and control of this syndrome. The specialist who best understands the upper airway must not escape this responsibility.

Dr. Michel Burihan Cahali

PhD. Assistant Physician – University of São Paulo Hospital and Servidor Público Estadual de São Paulo Hospital.

